# A “clip-and-snare” assisted endoscopic mucosal resection for an esophageal submucosal tumor

**DOI:** 10.1055/a-2361-1253

**Published:** 2024-09-04

**Authors:** Yiting Liu, Menghuan Zhu, Jian Gong, Jiajun Lu, Yagang Li

**Affiliations:** 1 74710 Department of Gastroenterology, First Affiliated Hospital of Dalian Medical University, Dalian, China

The clip-and-snare assisted endoscopic mucosal resection (CS-EMR) technique is a new and simple treatment option 1 . Here, we present a case of esophageal submucosal tumor resected with CS-EMR, which can easily remove the submucosal tumors completely ( Video 1 ).

A clip-and-snare assisted endoscopic mucosal resection (CS-EMR) technique for an esophageal submucosal tumor.Video 1

A 68-year-old man was referred for the endoscopic treatment of an esophageal submucosal tumor (6 mm). Gastroscopy showed a yellowish lesion. We used the transparent cap to cover the distal end of the endoscope, set a snare on the transparent cap, and inserted a clip into the channel in advance. The clip grasped the mucosa around the tumor, transforming the lesion into a “pedunculated polyp.” The snare was released and trapped the root of the lesion. The tumor was resected completely. The wound was closed by clips ( Fig. 1 ). Histological examination of the resected specimen revealed a submucosal spindle cell tumor. Pathological evaluation revealed a 0.7 × 0.6 × 0.6-cm mass; R0 resection was achieved.

**Fig. 1:**
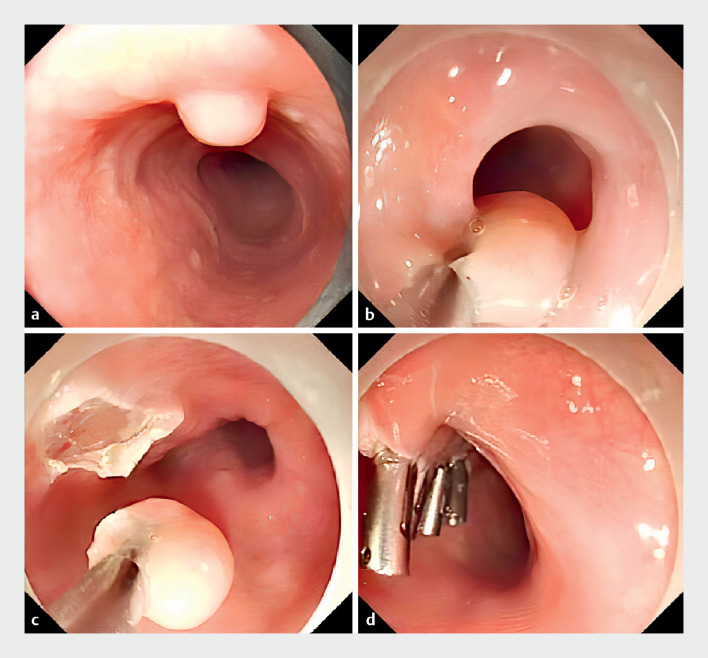
Endoscopic treatment of an esophageal submucosal tumor using the clip-and-snare assisted endoscopic mucosal resection (CS-EMR) technique. **a** Gastroscopy showed a yellowish lesion. **b** The snare was released and trapped the root of the lesion. **c** The tumor was resected completely. **d** The wound was closed by clips.

For esophageal submucosal tumors invading the submucosa, CS-EMR can be used to resect them efficiently and safely. We can choose different clip sizes depending on the size of the tumor. Therefore, CS-EMR can be one of the options for endoscopic resection of submucosal tumors of the esophagus.

Endoscopy_UCTN_Code_TTT_1AO_2AG_3AB

## Notes

Endoscopy E-Videos https://eref.thieme.de/e-videos
*E-Videos* is an open access online section of the journal *Endoscopy* , reporting on interesting cases and new techniques in gastroenterological endoscopy. All papers include a high-quality video and are published with a Creative Commons CC-BY license. Endoscopy E-Videos qualify for HINARI discounts and waivers and eligibility is automatically checked during the submission process. We grant 100% waivers to articles whose corresponding authors are based in Group A countries and 50% waivers to those who are based in Group B countries as classified by Research4Life (see: https://www.research4life.org/access/eligibility/ ). This section has its own submission website at https://mc.manuscriptcentral.com/e-videos .
